# Evaluation of Cold Chain Management Performance for Temperature-Sensitive Pharmaceuticals at Public Health Facilities Supplied by the Jimma Pharmaceuticals Supply Agency Hub, Southwest Ethiopia: Pharmaceuticals Logistic Management Perspective Using a Multicentered, Mixed-Method Approach

**DOI:** 10.1155/2021/5167858

**Published:** 2021-09-14

**Authors:** Diriba Feyisa, Awol Jemal, Temesgen Aferu, Fikadu Ejeta, Alem Endeshaw

**Affiliations:** ^1^Department of Pharmaceutics and Social Pharmacy, School of Pharmacy, College of Medicine and Health Sciences, Mizan-Tepi University, Mizan-Aman, Ethiopia; ^2^Department of Social and Administrative Pharmacy, School of Pharmacy, College of Health Sciences, Institute of Health, Jimma University, Jimma, Ethiopia; ^3^Pharmaceuticals Supply Agency, Jimma Hub, Jimma, Ethiopia; ^4^Department of Pharmaceutics and Social Pharmacy, School of Pharmacy, College of Medicine and Health Sciences, University of Gondar, Gondar, Ethiopia

## Abstract

**Background:**

Effective and efficient cold chain management maximizes utilization of healthcare resources, reduces cold chain products wastage, and improves the quality of health services. It eventually guarantees that clients receive cold chain products they need at service delivery points. The objective of this study was to evaluate cold chain management performance for temperature-sensitive medicines at public health facilities in Southwest Ethiopia supplied by the Jimma Pharmaceuticals Fund and Supply Agency hub. *Method and Materials*. The study used an institution-based cross-sectional study design. Forty-seven (47) public health facilities in Southwest Ethiopia were evaluated using checklists adopted from the Logistic Indicators Assessment Tool, Vaccine Management Assessment Tool, and Logistic System Assessment Tool.

**Results:**

The study revealed that the mean availability of essential cold chain products was 72.1 ± 14.8% while the average stock-out rate was 26.2 ± 8.6%. The median stock-out duration was 23 ± 21 days for all visited public health facilities. Two hundred and sixty-three (43.06 ± 15.3%) of the public health facilities' stock records were found accurate, and the wastage rate due to expiration was 9.2 ± 7.8% for all visited health facilities. Thirty public health facilities (63.8 ± 36.2%) had acceptable storage conditions. *Conclusions and Recommendations*. Supply chain performance at the study facilities was not adequate overall, and focused efforts need to be directed at managing the availability of critical cold chain medicines. Some cold chain management challenges demand the attention of the top management, while the rest can be addressed by operational management at the facilities through provision of appropriate training and supervision of the cold chain pharmaceutical handlers.

## 1. Introduction

Access to essential health services is crucial for improving the health of citizens [1]. The success of the essential health programs such as expanded program on immunization (EPI), reproductive, maternal, neonatal, child, and adolescent health services (RMNCAHS), diabetic care, and Human Immune Virus/Acquired Immune Deficiency Syndrome (HIV/AIDS) care services are dependent on effective cold chain management [2–5].

Temperature-sensitive pharmaceuticals are products of perishable nature and require beholding and distribution in a controlled environment. They include ergometrine, oxytocin, and insulin, vaccines, cold chain supplies, and diagnostic reagents (Rapid HIV test kit). Potency of these products may become less or even lost when exposed to temperature outside the recommended range. They need to be maintained between 2°C and 8°C temperature throughout the supply chain [6,7]. Cold chain management errors may lead to patient harm including unknown disease vulnerability requiring costly revaccination. Errors with vaccine use can also result in unintended and unrecognized vulnerability, leaving patients unprotected against severe disease [8].

Cold chain management is a means of warranting proper cold chain maintenance, a continuous and cohesive process to ensure availability and maintain the potency of temperature-sensitive pharmaceuticals [9]. Cold chain management plays a crucial role in ensuring the effectiveness of cold chain storage, handling, and stock management; rigorous temperature control in the cold chain; and adequate logistics management information systems [10]. Well-trained employees, dependable storage and temperature monitoring equipment, and precise cold chain medications' inventory management are the three essential aspects of a successful cold chain [11]. However, concerns over having adequate control in the cold chain are increasing in recent decades in the aftermath of an increasing volume and complexity of cold medicines and the complexity of the worldwide supply [12]. The rapid growth of biopharmaceutical business, complex global sourcing, and distribution chain has brought global interest in the supply chain management of cold pharmaceuticals [13].

Cold chain management is still a significant challenge in developing countries, and weaknesses are often observed during the transportation and storage of cold chain medicines [14]. Some factors contributing to weaknesses of the cold chain are delays during transportation, quality of refrigerators, a method of storage, too long storage at the health unit, improper use of refrigerators, power interruption, equipment breakage, and lack of trained personnel on cold chain management [15,16]. The same problems are being experienced in Ethiopia where cold chain medicines are losing their potency at the storage centers even if they arrive with their full potency [17,18].

The Pharmaceuticals Fund and Supply Agency (PFSA) is a legal entity to assure uninterrupted supply of medical equipment, medical supplies, diagnostic supplies, and medicines to the public health sector. It also manages the supply and logistics of temperature-sensitive health commodities [19]. The supply chain for temperature-sensitive pharmaceuticals in Ethiopia begins with the quarterly shipment of commodities from central storage to regional stores, zonal stores, and woredas and healthcare facilities. The regional and zonal stores are supplied quarterly from their respective upper-level store. The woredas and health facilities receive stock every month [20,21]. Pharmaceuticals logistics management activities such as quantification, procurement, inventory management, and logistic reporting must be evaluated and measured to improve the performance of health facilities [4,22–24].

Measurement of cold chain performance ensures a sustainable and reliable supply of cold chain products, resulting in high-quality healthcare services [25]. When analyzing the system's performance, qualitative evaluations such as good, fair, adequate, and poor are vague and difficult to utilize in any meaningful way. As a result, quantitative performance measures are often preferred. A numerical performance measure might be utilized when data are readily available [26]. It is imperative to measure cold chain supply performance to identify success, whether client requirements are met. Other purposes are to understand public health facility processes, identify problems/bottlenecks and product wastage management, make informed decisions based on facts rather than on supposition, and show whether logistic management performance improvements occurred. Measurement through crucial performance indicators offers consistency in measuring cold chain management performance at public health facilities [27].

Public Health Facilities (PHFs) in developing countries perform under unreliable electricity supply constraints, unreliable equipment, inadequate storage facilities, and improperly set up storage facilities. They also experience inadequate temperature monitoring, incorrect use of fridges at their service delivery point, weak validation system, and problems of frequent supervision and monitoring by a regulatory authority [28–31]. Ethiopia is not odd in this regard. Public health institutions offering health services in several administrative zones of the country including those in Southwest Ethiopia operate under unstable power sources and insufficient healthcare resources. Being in a temperate region with a hot and humid climatic condition, the temperature fluctuation in the Southwest Ethiopia brought temperature-sensitive pharmaceuticals to an extreme condition [32]. The current study evaluates the cold chain management performance, particularly pharmaceutical logistic management for temperature-sensitive medicines at PHFs supplied by the Jimma PFSA hub, Southwest Ethiopia.

## 2. Method

### 2.1. Study Area and Setting

The study was conducted at selected public health facilities in seven administrative zones and two special districts of the Southwest Ethiopia. Three of the zones included in the study, namely, Bunno-Bedele zone, Jimma zone, and Illu Aba-Bor zone, were from the Oromia region (southwest part) while four zones (Kaffa, Sheka, Dawuro, and Bench-Maji which is now split into Bench-Sheko and West Omo zone) and two special districts (Yem and Konta special districts) were from the Southern Nation, Nationalities and Peoples region. The area is among the most remote and underdeveloped places in the country and had an estimated population of 18–20 million. It is found in a tropical region with fewer clouds and more sun most of the season. Twenty-one public hospitals, 292 health centers, and 555 health posts supplied by the Jimma PFSA hub are rendering health services in the area currently [32].

### 2.2. Study Participants, Units, and Index Products

  Study participants and units: forty-seven public health facilities providing immunization services, reproductive, maternal, neonatal, and child health services (RMCHS), diabetic care, and ART services were selected for this study. Cold chain handlers who were working at immunization, pharmacy, and laboratory units of the selected public health facilities, one central EPSA, and one EPSA hub were the study participants, while logistic records and reports in the selected facilities were the study units.
  Study index products: eight WHO tracer vaccines, one ARV drug, one HIV test kit, one diabetic care medicine, and two WHO priority medicines for maternal health services were used as index products in the current study.

### 2.3. Study Design

Institution-based cross-sectional study design was employed using both quantitative and qualitative data collection techniques. Both primary and secondary data were collected to generate information on cold chain management performances for temperature-sensitive pharmaceuticals. A list of key temperature-sensitive pharmaceuticals and cold chain management performance indicators used to determine the management performance of the supply chain is presented as the supplementary file (supplementary Tables 1 and 2).

### 2.4. Sample Size Determination

For the quantitative study, the sample of health facilities required for this study was determined using the Logistic Indicators Assessment Tool (LIAT) prepared by the United States Agency for International Development (USAID)/DELIVER PROJECT. The LIAT document suggested that at least 15% of the target health facilities should be selected as a sample for conducting such study [33]. Accordingly, 47 hospitals and health centers (313*∗*15%) were selected as a study sample. The hospitals and health centers, one PFSA hub, and one central PFSA were selected by the random sampling technique. When it comes to the qualitative part, 16 key informants were determined of which 12 were from health facilities (one pharmacy unit head, one pharmacy store manager, one EPI focal person, and one hospital manager per hospital). Four hospitals were conveniently picked. At the same time, three individuals from the central PFSA directorate and a coordinator of cold chain supply from the Jimma PFSA hub were also interviewed purposively.

### 2.5. Sampling Technique

A clustered sampling technique was employed due to a heterogeneous nature of zones available in the study area. The administrative zones were used as clusters. The sample comprised health facilities selected from each zone based on the number of health facilities found in the respective zones (i.e., proportion to size). The study units were selected using a systematic random sampling technique based on the sampling frame for each zone. Hence, the 313 public health facilities had an equal and independent chance of being included in the selected sample. Accordingly, 47 health facilities were selected. Both health facilities and informant interviewee selection process are presented in the supplementary material (Supplementary Tables 3 and 4).

## 3. Data Collection Instrument, Source, and Collection Technique

The Logistic Indicator Assessment Tool (LIAT) and Vaccine Management Assessment Tool (VMAT) were adopted to develop a quantitative structured questionnaire. A standard qualitative open-ended questionnaire for key informant interviews was developed based on the Logistic System Assessment Tool (LSAT).

A predesigned and pretested observation checklist was used to collect detailed data on cold chain management of 8 WHO tracer vaccines, one ARV drug, one HIV test kit, and one diabetic care medicine. The same tool was used to collect data regarding two WHO priority medicines for maternal health service cold chain medicines including their acceptable storage condition, inventory control procedures, product availability, logistics management, and wastage. Six-month data (1 November 2016 to 31 April 2017) from bin cards were used to see the pattern of stock status in the health facilities. A checklist and desk reference review was utilized to determine cold chain products' availability and stock status. The calculations for these indicators used the formulas developed by the Management Sciences for Health.

A validated observation checklist designed according to the WHO guideline for storage of cold chain products was used to check the storage condition of cold chain products and to determine the status of standard cold chain management practice. Open-ended and closed semistructured questionnaire-based face-to-face interviews with key informants were used for qualitative data collection. The interviewer took notes, and the responses obtained for different questions were later transcribed.

### 3.1. Data Entry and Analysis

The quantitative data were checked for completeness, coded, entered into Epi-data version 3.1, and exported to SPSS version 22 for analysis. They were examined using descriptive statistics. A chi-square test was used to investigate the connection between variables, and a *p* value 0.05 at 95% confidence interval was considered statistically significant. Qualitative data were analyzed based on coding the thematic area.

### 3.2. Data Quality Assurance

Training was given to data collectors a head of the study. The tools used for data collection were also pretested. TA, FE, and AE reviewed tools for completeness and consistency, while DF with AJ oversaw data collection and regular activities.

### 3.3. Ethical Considerations

Ethical approval was obtained from the Ethics Review Board of the Institute of Health, Jimma University, reference no. IHRPGC/800/207. Both written and verbal consent from respondents was obtained before conducting the study. Participants were assured about confidentiality. Personal identifiers were not used to ensure the anonymity of respondents. The authors confirm that the study was carried out following the principles of the Declaration of Helsinki.

### 3.4. Operational Definitions

  Temperature-sensitive pharmaceuticals: any pharmaceutical good or product which, when not stored or transported within predefined environmental conditions and/or within predefined time limits, is degraded to the extent that it no longer performs as originally intended [34]. Eight WHO tracer vaccines (BCG, TT, OPV, Penta, measles, PCV, TAT, and Rabies) and 5 other United Nation life-saving commodities (khaletra, NPH insulin, rapid HIV test kit (Beijing Wentai), ergometrine, and oxytocin) were included in the evaluation.
  Good condition cold chain equipment: the cold chain equipment is intact and clean to keep vaccines within the required temperature range (2 C–8 C) [6,35].
  Good refrigerator storage: a refrigerator with adequate air circulation between the vaccine boxes, vaccine kept only on the refrigerator shelves and not in the door or bottom drawer, and no food or drink stored in the refrigerator.
  Acceptable/desirable/good storage and handling performance: percentage of public healthcare facilities that met more than 70% storage condition criteria/parameters of cold chain storage condition that needs to be met.
  Accurate stock record: public health facility record that was considered accurate if after adjusting for recent issues and receipts (within seven days), the record balanced with the physical count of the stock on the day of visit.
  Good product availability performance/adequate cold chain medicine stock:percentage of public healthcare facilities with stock available for more than 80% of products at the time of visit.
  Good inventory performance: performance generated by public health facilities with an accurate stock record, good stock maintenance, and good stock status.
  Temperature-sensitive medicine wastage at the PHF: the presence of sampled cold chain products damaged confirmed by vaccine vial monitoring (VVM), frozen confirmed by shake tests, expired by the date of usage at healthcare facilities that they are no longer safe to use as a result of short shelf life, improper forecasting leading to overstock, and above all, poor storage practice.
  Vaccine wastage rate: it was calculated using the formula (no. of doses wasted/ no. of doses issued) *X* 100 using data gathered from public health facilities on those variables in the formula. In contrast, the number of doses wasted was calculated using the formula no. of doses issued−no. of children benefitted [35].
  Vaccine wastage factor: it was calculated using the formula 100/(100−vaccine wastage rate) [35].
  High cold chain product wastage: when the product wastage of healthcare facilities is found to be above tolerable wastage ranges, above 25% of products were wasted.
  Good overall cold chain performance: the management performance of the supply chain is above the mean score/Z-score; public health facilities are said to be performing good when they achieved good performance in more than three of the five performance areas addressed by the study.

## 4. Results

### 4.1. General Information of Public Health Facilities

**Figure 1:**
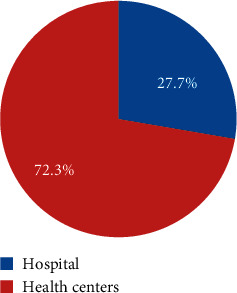
Type of public health facilities studied in Southwest Ethiopia, June 2017.

Thirty-five (72.3%) public health facilities were health centers (Figure 1).

### 4.2. Availability and Stock-Out of Temperature-Sensitive Pharmaceuticals (Product-Wise)

**Figure 2:**
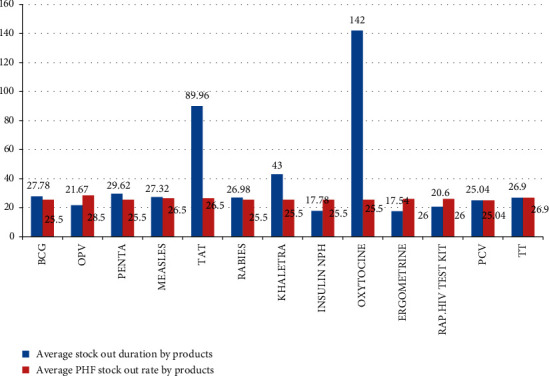
Average stock-out duration and stock-out rate by products at public health facilities in Southwest Ethiopia, June 2017.

The mean availability of key temperature-sensitive pharmaceuticals in all surveyed public health facilities was 72.1% ± 14.8%, while the median stock-out duration was 23.04 ± 21 days. OPV, PENTA, and BCG were the most available key temperature-sensitive biopharmaceuticals, while khaletra (lopinavir/ritonavir fixed-dose combination) and TAT were the least available pharmaceuticals (Figure 2 and Table 1).

### 4.3. Availability and Stock-Out of Key Temperature-Sensitive Pharmaceuticals per Type of Public Health Facilities

**Figure 3:**
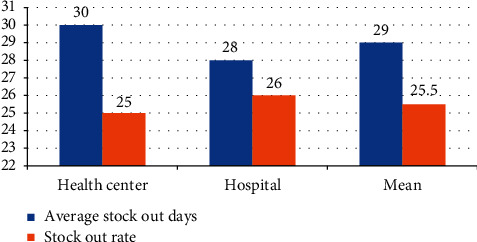
Average stock-out days and stock-out rate by facility type at public health facilities in Southwest Ethiopia, June 2017.

Average stock-out days of key temperature-sensitive pharmaceuticals among healthcare facilities were between 5 and 89 days. Hospitals registered 28 days, and health centers registered 30 days on average. The stock-out rate for the past six months before the data collection period was between 11% and 46%. The median stock-out rate of the key temperature-sensitive pharmaceuticals was 24.81% ± 8% for each healthcare facility, and the average stock-out rate was 26.2% ± 23% for each key temperature-sensitive pharmaceutical (Figures 2 and 3).

The health facilities recorded shortage and unfortunate stock-out of some essential Revolving Drug Fund (RDF) temperature-sensitive medicines that affected the delivery of healthcare to the client. An unforeseen increase in the number of patients to the health facilities and delays in payment of suppliers were some of the explored reasons. One key informant in charge of the pharmacy department of the surveyed hospital said “*…The procurement officer undertakes procurement by using the previous consumption of nonprogram cold chain medicines*. *Therefore*, *any unforeseen increase in the patient's attendance increases pressure on the available stock and causes a shortage of items and subsequent stock-out at our hospital…*.*”*

### 4.4. Inventory Management Performance at Study Public Health Facilities

**Figure 4:**
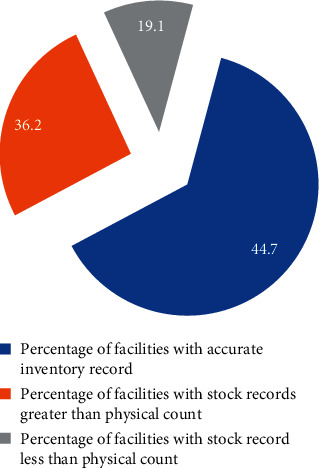
Stock record accuracy at public health facilities in Southwest Ethiopia, June 2017.

Out of 47 public health facilities, stock records reviewed in 21 (44.7%) were found to be accurate. On the other hand, less than one-third, 17 (36.2%), have shown a figure higher than the physical count. This study also revealed that the inventory record accuracy rate was between 25% and 100%. In comparison, the median inventory record accuracy rate was 39.2% while the average inventory record accuracy rate was 43.1% for each health facility (Figure 4).

Workload was asserted as a reason for not timely record keeping at public health facilities. A key informant at one visited hospital store said that *“…Getting to know the maximum level*, *minimum level, and order quantities of drugs involves many calculations*. *I end up not updating the stock cards because I have other obligations to attend….*”

Poor inventory management performance at health facilities was also responsible for causing shortage and stock-out of cold chain products. This was supported by one key informant saying “*…Unfortunately*, *some of the ministores of different units of the cold chain are not properly managed*, *while some departmental heads do not check stock levels regularly to report shortages early*. *The inability to monitor stock level causes total depletion of certain goods that subsequently results in cumulative stock-out and affects healthcare delivery at our hospital….”*

### 4.5. Logistic Management Information System (LMIS) Performance at Study Public Health Facilities

**Figure 5:**
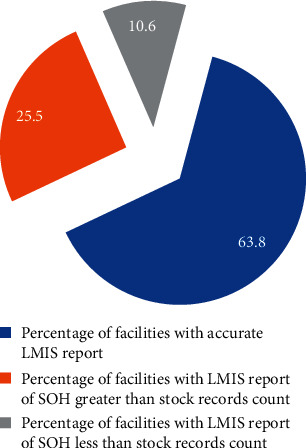
LMIS report accuracy at public health facilities in Southwest Ethiopia, June 2017.

Out of 47 public health facilities, the LMIS report reviewed in 30 (63.8%) were found accurate. On the other hand, less than one-third, 12 (25.5%), have shown a count figure of LMIS report higher than stock record count. This study found that the inventory record accuracy rate ranges between 25% and 100%. The median LMIS report accuracy rate was 32.5%, while the average LMIS report accuracy rate was 53.3% for each health facility (Figure 5).

High workload was one of the reasons the health facilities were underperforming in logistic management operations. The store manager of one of the surveyed hospitals stated that *“…Since my fellow departed*, *I am all alone for this hospital's store management*. *Managing special commodities such as cold chain medicine is always difficult*: *a lot of my effort is spent on visual assessment of product quality such as integrity of products*, *whether they got frozen or expired (VVM unacceptable), and physical counts to verify the number on the invoice*; *keeping the status of these items by updating bin cards to track their movements and register loss*, *damage*, *and issues on these records is very challenging….”*

### 4.6. Temperature-Sensitive Pharmaceutical Storage Performance at Public Health Facilities

The storage criterion that met most by the public health facilities studied was the presence of standard cold chain refrigerators and freezers, 65 (85.5%), refrigerator on recommended temperature range, 40 (90.9%), while the storage criterion that was met least was the presence of standby power supply, 20 (42.5%) (Table 2).

**Figure 6:**
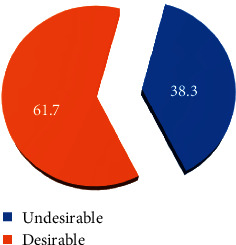
Storage performances for temperature-sensitive pharmaceuticals at public health facilities in Southwest Ethiopia, June 2017.

Public health facilities were assessed based on 18 principles. The public health facility's storage performances were regarded as acceptable or unacceptable cold chain storage. Out of 47 public health facilities, 29 (61.7%) had acceptable cold chain storage conditions (Figure 6).

Qualitative study undertaken to explore the reasons that contributed to undesirable store management performance came up with different findings. Most key informants said inadequate in-service training on cold chain management, storage and handling of cold chain medicines, and record keeping to be the most common reasons. High workload was also among the explored reasons why health facilities were underperforming in storage management operations. The store manager of one of the surveyed hospitals stated that *“…Yes*, *I got a piece of training including EPI management with my departed fellow*. *But, I am all alone now and responsible for the entire hospital's store management*. *As you see today*, *I have not even checked refrigerators and updated temperature charts on those refrigerators….”*

### 4.7. Temperature-Sensitive Pharmaceutical Wastage and Expiry Management in the Surveyed Health Facilities

Product expiry was observed in more than half of the hospitals (53.8%) and around one-third of health centers visited (29.4%) during the assessment period. The expiry was high at the hospitals than at health centers. Wastage rate due to expiry was 9.2 with a standard deviation of 8.9 for all study public health facilities. Purchases were made in bulk in anticipation of the NPD cold chain price increase, but sometimes, close to half of the items would expire. A supply officer stated, *“…for RDF temperature-sensitive pharmaceuticals*, *before*, *like two or a year ago*, *we used to procure in large quantities because we perceived price increases shortly*. *Certain times*, *close to half of the quantity expires/gets wasted….”*

The pharmacy unit head and facility store managers disclosed that there are times when the regional PFSA hub compels the facilities to push items that are unneeded and close to expiry. The head of a pharmacy unit in one hospital lamented that *“…the Jimma pharmaceutical supply agency hub also does push revolving drug fund cold chain goods to our hospital*, *and we have no choice than to collect them*. *These goods are sometimes not to our specifications and needs that the products will go unused and, therefore, later expire*....*”*

Six vaccines (BCG, OPV, DPT, TT, Measles, and PCV) that were given to children resulted in an unacceptable wastage rate and wastage factor (WF) (Table 3). The wastage factor and wastage rate for BCG were found the highest with 2.32 and 57% followed by PCV, 1.35 and 26% among liquid and injectable forms of vaccines. The liquid, lyophilized form of oral OPV registered the highest wastage factor, 1.61, and wastage rate, 38%. The inability to strictly adhere to the Joint WHO-FMOH multidose open vial policy and undesirable storage practice of multidose vial vaccine were among the main reasons for vaccine wastage at surveyed public health facilities. This was justified by one immunization focal person saying *“…Unnecessary discard of the vial was observed*. *Some vaccine providers discard vaccines after a single use in this immunization room without bothering to assess whether the vaccine vial submerges*, *whether the vaccine vial motor has reached its discard point, and whether the expiry date has not passed*. *While others discard a reconstituted vial without waiting for either a quarter of a day after it is opened or another immunization session within the same day…”*. Another informant stated *“…opened vial often returns to the refrigerator with no label on them about their first opened date*; *we have no choice but discard them solely due uncertainty on when to discard them resulting in increment on vaccine wastage….”*

### 4.8. Summary of Overall Supply Chain Management Performance for Temperature-Sensitive Pharmaceuticals

A significant sum of PHFs were practicing desirable storage conditions (63.8%) and accurate LMIS reports (63.1%), while some had poor/inaccurate stock records (56.9%) and high product wastage (55.3%) (Table 4).

### 4.9. Challenges Faced by Public Health Facilities in Managing Temperature-Sensitive Pharmaceuticals

The main challenges experienced were inadequate budget for cold chain equipment maintenance, electric power interruption, shortage of cold chain equipment, and lack of timely maintenance. With regard to budget shortage, one key informant stated that *“…as to me, one of the barriers that come to me at this moment in time is the shortage of budget for effective vaccines' cold chain equipment maintenance*. *Besides*, *I believe the management support is also not to a level I have expected*, *we need to be proactive in such a situation….*”

Limited training for cold chain handlers was blamed to hamper health facilities' performances. For instance, an EPI focal person at one hospital noted, *“*...*I have not had the opportunity to receive training on logistics management*, *let alone on cold chain management*. *I have never been monitored*, *and I usually create and submit a list of out-of-stock items to the in charge and wait….”*

Lack of cold chain guideline and its utilization (where the guideline was available) for cold chain management at some hospitals was also brought up as a problem in acquiring supplies. “*…We do not have guidelines to follow order processing and buying nonprogram cold chain pharmaceuticals and overall management of cold chain commodity level at this hospital…,”* a pharmacist in charge at one hospital said.

### 4.10. Proposed Solutions for Overcoming Challenges and Ensuring Effective Temperature-Sensitive Pharmaceutical Management: Key Informants' Suggestions

Most of the key informants agreed that capacity building, distribution of cold chain equipment with adequate spare parts, solving electric power interruption, and strict adherence to cold chain management manual should be carried out to minimize cold chain management problems. Others suggested the way to achieve properly performing cold chain management operations; some outlined how to improve temperature-sensitive pharmaceutical storing, handling, and management. One key informant stated, *“…putting cold chain medicines in a circle on an icepack compromises cold chain as it may cause freezing of temperature-sensitive medicines*. *Due to careless in storing*, *handling*, *and administering*, *these products may lose their potency at the end*....*”*

The availability of cold chain equipment such as fridge tags and solar refrigerators was deemed critical for successful vaccine cold chain management. One key informant supported this issue pointing *“*... *availability of fridge tags that monitor temperature continuously and report out-of-range temperatures is a good practice on vaccines' cold chain management and this practice needs to be scaled up.*”

Another key informant described that *“…availability of the solar fridge is a good practice*. *Since it does not need electric power/kerosene and has self-adjustable constant temperature*, *it maintains temperature-sensitive medicines at a recommended temperature at any time….”*

The other key informant suggested special consideration for handling and managing cold chain pharmaceuticals stating that *“*...*cold chain health commodities' potency and integrity are kept well*. *The cautious follow-up is crucial due to their integral fragility and sensitivity to an extreme temperature range at both ends*. *We need to care for these products in a special way as they are products of high public health importance and endure positive externalities to the community*….”

Setting proper information flow and building smooth relationship with the surrounding health facilities and suppliers ensure interdepartmental and facility reliance on one another in times of need. A pharmacist in charge at one hospital stated that, *“…In situations of shortage*, *we fall on the shoulder of other facilities that may have enough goods at their disposal*. *We do not halt the provision of our services because of shortage*. *We*, *however*, *ensure we return these supplies to the facilities whenever they are available at the stores….”*

The supply coordinator in charge of one surveyed hospital stressed the importance of proper information flow. He said “…*Information flow among departments enables us to borrow from other facilities in times of shortages*. *Most of the time*, *I call my professional fellows in other health facilities to find out if they have enough stock and I can borrow from them*. *I enjoy the information flow in the organization since it facilitates smooth performance in terms of service delivery….”*

## 5. Discussion

Cold chain management performance has not been extensively evaluated in Ethiopia similar to many developing countries. Therefore, the current study is believed to give some insights into and will help to improve the cold chain management performances at public health facilities in the study area and other regions with similar characteristics to the studied facilities.

The primary reason for holding stock in the pharmaceuticals supply system is to ensure the availability of essential items almost all the time. The mean availability of temperature-sensitive medicines in public health facilities in the current study was 72.1%. This finding corroborates a nationwide survey of the pharmaceutical sector in Ethiopia, Dar es Salaam, and Tanzania's mainland [36–38]. Nevertheless, this finding is not adequate as mean availability should be over 80% [39].

The average duration that the key temperature-sensitive pharmaceuticals are out of stock indicates the capacity of a system to maintain constant supply of products over time. The median stock-out duration was 23.04 days for the health facilities while the average stock-out duration was 29.30 days for each public health facility in the current study. These results show the logistics system in public health facilities in Southwest Ethiopian region (SWE) supplied by the Jimma EPSA hub was not performing well. Such conclusion is partially made on the basis that the median stock-out duration should ideally be zero [39]. The current finding is higher than that of a study conducted on Tanzania's mainland which revealed the average stock-out duration of the same medicines in public health facilities to be 15 days [37]. Maintaining temperature-sensitive pharmaceutical stockpiles at the appropriate level was also a challenge; according to a study conducted in Dar es Salaam, around 20% of tracer medicines were out of stock, with an average stock-out time of 8 days in three district hospitals [37].

Accurate inventory record keeping is essential for proper inventory management at all levels of public facilities. Inaccurate recording increases the risk of stock-outs, leaks, and expiry [40]. This study found that all health facilities, regardless of the facility type, had incorrect stock record keeping. This result agrees with findings from healthcare facilities of Dar es Salaam [38] and Jordan [40] where stock-out and inventory management problems were reflected by stock records' accuracy. Despite this numerical difference, the issue of inaccurate stock records is critical. Although the ideal target for this indicator is 100%, most SWE public health facilities were not performing well from the target perspective. Qualitative findings highlighted that increased workload was the primary cause for delay and subsequent forget in recording issues and receipts transactions as they become due.

Cold chain medicine wastage indicates the inefficiency of logistics system management performance. Qualitative findings in this regard indicated lack of proper documentation when moving expired items and poor practice of dealing with discarded old and damaged products. Vaccines such as BCG, Measles, and OPV were wasted the most followed by TAT and oxytocin in some public healthcare facilities. The unusable stock of NPH insulin was also found in other public healthcare facilities visited. One of the main reasons for the wastage of vaccines at public health facilities was the temperature sensitivity attributed to frequent power outage.

Proper storage is vital to maintain the purity, potency, safety, and effectiveness of cold chain medicines [39]. Cold chain storage condition performances in the study facilities were not impressive as only slightly above half had acceptable storage conditions. Most of the healthcare facilities visited had standard and functional cold chain refrigerators. They also kept their vaccine refrigerators at temperature of +2^0^C to +8^0^C at the time of visit similar to findings from the northwest region of Cameroon [28], Central Ethiopia [31], Chandigarh [41], Philippines [42], Southern Nigeria [43], South India [44], and Northwest Cameroon [14]. However, this finding is contrary to a study conducted in India that reported 73% of the cold chain equipment to be available and maintain the required temperature [45]. The difference might be explained by the variation in the level of knowledge, motivation and adequacy of supervision of cold chain handlers, and cold chain handlers' level of exposure to formal training on cold chain management. The primary reasons for not maintaining temperature within acceptable range were lack of consistent power supply, frequent power outages, and equipment failure.

Slightly less than two-thirds of the visited healthcare facilities' stores had good arrangement practices. Around one-tenth of them were found using domestic refrigerators for vaccines storage. Around one-third of public health facilities on average had stored other items such as fruits and water with temperature-sensitive medicines in refrigerators. This storage practice is improper and not recommended at all. This finding is similar to a study from the coast region of Tanzania [39]. Expired and frozen vaccines were present in some healthcare facilities in the current study similar to the results from the coastal region of Tanzania [37], urban health centers of the metro city of India [46], northwest region of Cameroon [14], and the Philippines [42].

Poor storage conditions of vaccines on transit and in public healthcare facilities, nonadherence to good storage practices, and shortage of service providers at some public health facilities were among the problems found in the current study. Infrastructure-related issues such as lack of reliable transportation, unreliable electricity, and lack of alternative power sources during electricity outage and poor quantification and forecasting were also found hindering proper management of cold chain products similar to studies from India [47], Mozambique [48], and Nigeria [49].The absence of standby generators and lack of fuel (for the available and functioning standby generators) combined with irregular and extremely unpredictable power supply substantially increases cold chain thawing and accelerates loss of potent products.

## 6. Conclusions

The availability of key temperature-sensitive pharmaceuticals in public health facilities was inadequate. A majority of public health facilities were inefficient as temperature-sensitive pharmaceutical wastage was high. A significant sum of public health facilities failed to meet desirable storage conditions for the studied products. An unreliable power supply appears to be the main problem in maintaining the recommended temperature range for proper cold chain storage. Adherence to good storage practices also needs attention from cold chain handlers. Overall, supply chain performance of the studied facilities was not adequate and budget constraints require attention of the top management.

## 7. Recommendations

District health administrators can better provide comprehensive training on record keeping and cold chain storage management at a reasonable interval to cold chain handlers. Chief executive directors (CEDs) of hospitals and directors of health centers are required to undertake internal supervision of their respective health facilities. Store managers need to monitor their stock of temperature-sensitive pharmaceutics daily through updating stock cards, registers, and LMIS reports. Supply chain coordinators ought to review the stocks delivered against the minimum stock levels before ordering temperature-sensitive pharmaceuticals. Cold chain handlers also need to reduce freezing and stock-out of cold chain medicines by utilizing effective logistics and inventory management techniques while delivering and receiving temperature-sensitive pharmaceuticals.

## 8. Study Limitations and Areas for Further Research

Due to the short evaluation period (six months and on the spot), this study could only represent the current availability of temperature-sensitive medicines and did not indicate changes in the availability in the study area. The findings of this study and their application are limited to the public healthcare facilities surveyed in SWE, not the entire country. More meaningful results would have been achieved had the scope of the study been broadened to cover the performance of cold chain management at public health facilities across the country. Further research has to be carried out to develop comprehensive findings that can be generalized to the country.

The key areas of cold chain management performance were equally weighted when estimating the overall supply performance for key cold chain products. At the same time, they might not have the same effect on the health facility's overall performance. Still, all elements of the performance were appreciated and the scoring used was validated. However, the study showed exciting results to improve cold chain management performance for key temperature-sensitive pharmaceuticals at public health facilities supplied by the Jimma PFSA hub, Southwest Ethiopia.

## Figures and Tables

**Table 1:** Mean availability by product at public health facilities in Southwest Ethiopia, June 2017 (*N* = 47).

Key temperature-sensitive pharmaceuticals	Availability per health facility (mean ± SD)
BCG	91.8 ± 8.2%
OPV	93.2 ± 6.8%
PENTA	93.2 ± 6.8%
MEASLES	90.9 ± 9.1%
TAT	35 ± 24.7%
PCV	40.4 ± 40.4%
BEIGING WENTAI	43.7 ± 39.8%
ERGOMETRINE	33.1 ± 30.4%
OXYTOCINE	84.1 ± 25.9%
INSULIN-NPH	47.3 ± 43.8%
ANTI-RABIES	65.4 ± 19.4%
TT	87.2 ± 12.8%
KHALETRA	14.9 ± 23.9%
MEAN AVAILIBILTY	72.1 ± 23.5%

**Table 2:** Public healthcare facilities fulfilling a specific storage criterion in Southwest Ethiopia, June 2017 (*N* = 47).

Storage condition parameters	Frequency (%)
Good arrangement inside refrigerators (with total refrigerators = 44)	29 (65.9%)
Presence of a freezer tag monitor in the freezer (with total freezers = 32)	22 (68.7%)
Daily monitoring and recording of temperature in chart	26 (55.3%)
Presence of trays which are used for arrangement	29 (65.9%)
No mixture of other items such as fruits and water with cold chain medicine in the refrigerator	33 (75%)
Presence of good storage space for the refrigerator	28 (63.6%)
Cold chain medicine freezer storage capacity adequate to accommodate all stocks	23 (71.9%)
Presence of standard cold chain medicine refrigerators and freezers (*n* = 76)	65 (85.5%)
Cold chain medicine are not found stored in the domestic freezer (*n* = 47)	31 (65.9%)
Presence of standby power supply	20 (42.5%)
Refrigerator maintained at the recommended temperature range (+2°C to +8°C)	40 (90.9%)
Presence of a working thermometer inside refrigerators	23 (52.3%)
Quantification performed during ordering	41 (87.2%)
Cold chain products freezer maintained at a temperature range of −15°C to −25°c	28 (87.5%)
Correct placement of icepacks in the freezer (*n* = 32)	21 (65.6%)
Cold chain products are not found frozen in refrigerators and freezers (*n* = 76)	66 (86.8%)
No expired cold chain products at the health facility at the time of observation	31 (65.6%)
No cold chain products stored on the refrigerator door shelf	26 28(59.1%)

**Table 3:** Wastage rate of WHO tracer vaccines at public health facilities in Southwest Ethiopia, June 2017 (*N* = 47).

Vaccine's name	Number of doses in a vial vaccine	Doses used	Number of children vaccinated	Doses wasted	Wastage rate(WR) (%)	Wastage factor (WF)
BCG vaccine	10- dose vial	7826	3352	4474	57	2.32
Oral polio vaccine	20- dose vial	12345	7652	4693	38	1.61
Penta vaccine	10- dose vial	14536	10854	3682	25	1.33
Measles vaccine	5- dose vial	4289	3479	810	19	1.23
TT	10- dose vial	6059	3984	2075	34	1.52
PCV	10- dose vial	5182	3847	1335	26	1.35

**Table 4:** Summary of overall cold chain management performance for key temperature-sensitive pharmaceuticals at public health facilities in Southwest Ethiopia, June 2017 (*N* = 47).

Summary characteristics	Performance category	Frequency(%)
PHF product availability	Good/adequate stock	17 (36.2%)
	Poor/inadequate stock	30 (63.8%)

PHF inventory recording and control	Good/accurate stock records	20 (42.6%)
	Poor/inaccurate stock records	27 (57.4%)

PHF storage and handling	Good/desirable storage condition	30 (63.8%)
	Poor/undesirable storage condition	17 (36.2%)

Product wastage management	Good/low product wastage	21(44.7%
	Poor/high product wastage	26 (55.3%)

LMIS performance	Good/accurate LMIS reports	30 (63.8%)
	Poor/inaccurate LMIS reports	17 (36.2%)

Overall supply management performance	Good	20 (42.6%)
	Poor	27 (57.4%)

## Data Availability

The datasets generated and/or analyzed during the present study are available from the corresponding author on reasonable request.

## Acronyms and Abbreviations

BCG: *Bacillus* Chalmette Guerin
DPT-Hep-B-Hib: Diphtheria, Pertussis, Tetanus, Hepatitis B, and Influenza type *b*
COR: Crude odds ratio
CED: Chief executive director
EPI: Expanded program on immunization
EEFO: Early expiry first out
FEFO: First to expiry first out
FIFO: First in first out
FMOH: Federal Ministry of Health
KIIG: Key informant interview guide
LMIS: Logistic Management Information System
MSH: Management Sciences for Health
LIAT: Logistic Indicators Assessment Tool
LSAT: Logistic System Assessment Tool
OPV: Oral polioviruses vaccine
PFSA: Pharmaceutical Fund and Supply Agency
PHF: Public health facility
RMNCAHS: Reproductive, maternal, neonatal, child, and adolescent health services
TT: Tetanus toxoid vaccine
UNICEF: United Nations Children's Fund
VMAT: Vaccine Management Assessment Tool
VVM: Vaccine vial monitoring
WHO: World Health Organization.

## Supplementary Materials

Supplementary Materials Supplementary Table 1: list of 13 key temperature sensitive medicines (8-WHO tracer vaccines and 5 other global lifesaving temperature sensitive medicines) at public health facilities in Southwest Ethiopia, June 2017. Supplementary Table 2: indicator used to evaluate supply chain performances of cold chain products at public health facilities in Southwest Ethiopia, June 2017. Supplementary Table 3: distribution of public health facilities in different zones of two Ethiopian regional states supplied by the Jimma Pharmaceuticals Fund and Supply agency (PFSA) hub, Southwest Ethiopia, June 2017. Supplementary Table 4: sampling strategy for key informant interviews at public health facilities in Southwest Ethiopia, June 2017.
