# Supplementary material for: Evaluation of Cold Chain Management Performance for Temperature-Sensitive Pharmaceuticals at Public Health Facilities Supplied by the Jimma Pharmaceuticals Supply Agency Hub, Southwest Ethiopia: Pharmaceuticals Logistic Management Perspective Using a Multicentered, Mixed-Method Approach
Source: Adv Pharmacol Pharm Sci. 2021 Sep 14;2021:5167858. doi: 10.1155/2021/5167858 (PMC8455189; doi:10.1155/2021/5167858)
Supplement: Supplementary Materials — Supplementary Table 1: list of 13 key temperature sensitive medicines (8-WHO tracer vaccines and 5 other global lifesaving temperature sensitive medicines) at public health facilities in Southwest Ethiopia, June 2017. Supplementary Table 2: indicator used to evaluate supply chain performances of cold chain products at public health facilities in Southwest Ethiopia, June 2017. Supplementary Table 3: distribution of public health facilities in different zones of two Ethiopian regional states supplied by the Jimma Pharmaceuticals Fund and Supply agency (PFSA) hub, Southwest Ethiopia, June 2017. Supplementary Table 4: sampling strategy for key informant interviews at public health facilities in Southwest Ethiopia, June 2017. [file 5167858.f1.pdf]

List of supplementary Tables and Figures

Supplementary Table 1: - Lists of 13 Essential temperature sensitive medicines (8-WHO tracer vaccine and 5-other global lifesaving temperature sensitive medicines), June, 2017

S/N	Key cold chain products	NO. of doses in vial
1	BCG vaccine	10 –dose vial vaccine
2	OPV	20 –dose vial vaccine
3	Penta vaccine	10 –dose vial vaccine
4	Measles vaccine	5 –dose vial vaccine
5	TAT	10 –dose vial vaccine
6	Rabies vaccine	1-vial vaccine
7	Khaletra(lopinavir /retinovir) fixed dose combination)	1-vial
8	Insulin (NPH)	1-vial
9	Oxytocine	1-vial
10	Ergometrine	1-vial
11	HIV rapid test kit(BWT(Beiging Wentai))	1-vial
12	TT vaccine	10 –dose vial vaccine
13	PCV	10 –dose vial vaccine

Supplementary Table 2:-Indicator used to evaluate supply chain performances of cold chain products at public health facilities in southwest Ethiopia, June 2017

**Indicator used for the present study**			
**S/n**	**Indicator**	**Definition**	**Formula**
1	% of PHFs with temperature medicines available	Tells number of PHF with adequately available key cold chain products from study public health facilities	(Number of public health facilities that have stock continuously available at a time of visit for >= 80% of key cold chain products /47)*100
2	Average stock out duration	Tells duration for which key cold chain products were out of stock at each PHF	(Sum of stock out days /13)
3	stock out rate	Tells the rate at which key cold chain products were out of stock at study public health facilities	(No. of health facilities assessed that experienced a stock out of key cold chain products/47)*100
4	Inventory record accuracy rate	Measures accuracy of stock balances in stock ledger(card) over range of key cold chain products	(Number of item where stock record count equals to physical stock count/13)*100
5	% of PHFs that had accurate LMIS reports	Measures percentage of PHFs having no discrepancy between balance stated on the stock card and the LMIS reports	(number of public health facilities with no discrepancies/47)*100
6	Wastage rate	Measures % of stock of key cold chain product s wasted (unusable) during last one year	(Unusable stock quantity key cold chain products over one year period /total stock quantity of key cold chain products over one year period)*100
7	% of PHF with acceptable storage condition	Measures percentage of PHFs that fulfilled at least 80% storage condition defined in checklist for evaluation of cold chain storage unit	(Number of public health facilities storage unit that fulfilled 80% of storage condition /47)*100

Supplementary Table 3:- The distribution of public health facilities in different zones of two Ethiopian regional states supplied by Jimma Pharmaceutical fund and supply agency (PFSA) Hub, June, 2017)

S/n.	Regional states	Zones districts	number of health facilities	proportional sample size of health facilities
1	Ooromiya regional state	Jimma zones' districts	133	20
2		I/Ababorzones' districts	42	6
3		Buno-Bedelezones' districts	27	4
4	Southern nation, nationalities and peoples regional state	Kaffazones' districts	47	7
5		Bench-Maji zones' districts	40	6
6		Shekazones' districts	14	2
7		Konta zones' districts	4	1
8		Yem specialworeda	6	1
Total			313	47

Supplementary Table 4:- The sampling strategy for Key informant interviews for the present study (2017)

**Institution**		**Selected site**	**Selected KI**	**Total**
Public health faciliti es	hospitals	Jimma zone [Agaro general hospital]	One CEO, Store manager and EPI Focal person per each hospitals	4
		I/Ababor zone [Metu karl hospital]		4
		Kaffa zone [Gabra tsadik shawo hospital]		4
PFSA	Centeral PFSA	Pharmaceutical Forecasting and capacity building directorate	Directors/responsib le managers/	1
		Cold room management unit		1
	Jimma PFSA hub	Distribution and storage directorate		1
